# Associations of the uric acid related genetic variants in *SLC2A9* and *ABCG2* loci with coronary heart disease risk

**DOI:** 10.1186/s12863-015-0162-7

**Published:** 2015-01-30

**Authors:** Xu Han, Lixuan Gui, Bing Liu, Jing Wang, Yaru Li, Xiayun Dai, Jun Li, Binyao Yang, Gaokun Qiu, Jing Feng, Xiaomin Zhang, Tangchun Wu, Meian He

**Affiliations:** Institute of Occupational Medicine and the Ministry of Education Key Lab of Environment and Health, School of Public Health, Huazhong University of Science and Technology, Wuhan, China; MOE Key Lab of Environment and Health, School of Public Health, Tongji Medical College, Huazhong University of Science & Technology, 13 Hangkong Rd, Wuhan, Hubei 430030 China

**Keywords:** Coronary heart disease, Uric acid, Polymorphism, Gene-environment interaction

## Abstract

**Background:**

Multiple studies investigated the associations between serum uric acid and coronary heart disease (CHD) risk. However, further investigations still remain to be carried out to determine whether there exists a causal relationship between them. We aim to explore the associations between genetic variants in uric acid related loci of *SLC2A9* and *ABCG2* and CHD risk in a Chinese population.

**Results:**

A case–control study including 1,146 CHD cases and 1,146 controls was conducted. Association analysis between two uric acid related variants (SNP rs11722228 in *SLC2A9* and rs4148152 in *ABCG2*) and CHD risk was performed by logistic regression model. Adjusted odds ratios (ORs) with 95% confidence intervals (CIs) were calculated. Compared with subjects with A allele of rs4148152, those with G allele had a decreased CHD risk and the association remained significant in a multivariate model. However, it altered to null when BMI was added into the model. No significant association was observed between rs11722228 and CHD risk. The distribution of CHD risk factors was not significantly different among different genotypes of both SNPs. Among subjects who did not consume alcohol, the G allele of rs4148152 showed a moderate protective effect. However, no significant interactions were observed between SNP by CHD risk factors on CHD risk.

**Conclusions:**

There might be no association between the two uric acid related SNPs with CHD risk. Further studies were warranted to validate these results.

**Electronic supplementary material:**

The online version of this article (doi:10.1186/s12863-015-0162-7) contains supplementary material, which is available to authorized users.

## Background

Coronary heart disease (CHD) is one of the leading causes of morbidity and mortality throughout the world [1]. The World Health Organization estimated that each year more than 700,000 people die from CHD in China with a substantial economic burden [2]. CHD is a multifactorial disease resulting from genetic, environmental factors and their interaction [3]. Known risk factors for CHD include obesity, smoking, diabetes, dislipidemia and etc. [4-7].

Uric acid, as the end product of purine metabolism, is a major cause of gout [8,9]. Studies indicated that uric acid levels were associated with insulin resistance [10] and metabolic syndromes [11]. In addition, epidemiological studies have investigated the association of uric acid levels with CHD risk with inconsistent results [4,12-19]. Some studies found uric acid levels were positively associated with CHD risk [4,13,14,20]. In contrast, some studies found no association between them [21,22]. Similar controversial findings were found in Chinese population [23-25]. Therefore, it still remains to be investigated whether there is a causal association between serum uric acid levels and CHD risk.

Recent genome-wide association studies (GWASs) identified multiple genetic loci associated with serum uric acid concentrations [9,13,18,26,27]. Our previous GWAS also confirmed two reported loci *SLC2A9* (solute carrier family 2, facilitated glucose transporter member 9, 4p16.1) [18,22,26,28,29] and *ABCG2* (ATP-binding cassette, sub-family G, member 2, 4q22) [12,30,31] positively associated with serum uric acid levels in a Chinese population [32]. Several studies investigated the associations between the genetic variants in the uric acid related loci of *SLC2A9* and *ABCG2* and the risk of CHD among Europeans [12,22,25,33,34] and found no association between them. It is necessary to further investigate their associations among other populations.

In the present study, a case–control design (consisting of 1,146 cases with 1,146 age- and sex- frequency matched controls) was adopted and two SNPs rs11722228 (intron in *SLC2A9*) and rs4148152 (intron in *ABCG2*), which were in the previously reported uric acid related loci and confirmed in our GWAS [32,35,36], were selected to examine their associations with the CHD risk among Chinese. To our best knowledge, there were no studies investigating the association of these two SNPs and CHD risk before. The results in the present study will help us to verify the existence of causal relationship between serum uric acid levels and CHD risk.

## Methods

### Study population

The subjects included in the present study were recruited consecutively at the department of cardiology from three hospitals (Tongji Hospital, Union Hospital, and Wugang Hospital) in Wuhan city (Hubei province, China) between May 2004 and October 2006. Quantitative coronary angiography was performed by experienced cardiologists who had no knowledge of the patients’ clinical information. After exclusion of those who had acute renal and liver diseases or with incomplete information, 2,292 individuals of 1,146 cases with 1,146 age- and sex- frequency matched controls were included in our study. All the subjects were unrelated Chinese Han individuals and lived in Hubei province, the central China. The baseline characteristics of cases and controls were shown in Table 1. Our study has been approved by the Medical Ethics Committee of the School of Public Health, Tongji Medical College. Written informed consents were obtained from all the participants.

**Table 1:** **Baseline characteristics of the CHD case and control subjects**

**Variables**	**CHD cases (n = 1,146)**	**Control subjects (n = 1,146)**	***P*** **value**
Age (years)	60.0 ± 10.3	60.5 ± 11.3	0.229
Gender (male/female)	891/255 (77.7/22.3)	901/245 (78.6/21.4)	0.613
BMI (kg/m^2^)	24.4 ± 3.3	23.7 ± 3.1	<0.01
Smoking, no/yes, (%)	774/369 (66.7/32.3)	686/460 (59.9/40.1)	<0.01
Drinking, no/yes, (%)	822/317 (72.2/27.8)	776/365 (68.0/32.0)	<0.01
Systolic blood pressure (mmHg)	136.0 ± 25.3	133.6 ± 29.0	0.034
Diastolic blood pressure (mmHg)	82.9 ± 15.2	82.0 ± 11.3	0.110
Fasting blood glucose (mmol/L)	6.2 ± 3.0	5.6 ± 2.2	<0.01
Total cholesterol (mmol/L)	4.4 ± 1.1	4.7 ± 0.9	<0.01
Triglyceride (mmol/L)	1.7 ± 1.4	1.6 ± 1.3	0.283
HDL cholesterol (mmol/L)	1.2 ± 0.7	1.1 ± 0.4	<0.01
LDL cholesterol (mmol/L)	2.6 ± 0.9	2.7 ± 0.8	<0.01
Past history			
Diabetes, no/yes, (%)	824/314 (72.4/27.6)	1080/65 (94.3/5.7)	<0. 01
Hypertension, no/yes, (%)	351/790 (30.8/69.2)	784/361 (68.5/31.5)	<0. 01
Family history of CHD, no/yes, (%)	947/158 (85.7/14.3)	1133/10 (99.1/0.9)	<0.01

Values are mean ± SD, n (%) or as indicated. CHD: coronary heart disease; BMI: body mass index; HDL: high density lipoprotein; LDL: low density lipoprotein.

### Data collection

General health examination was performed including standing height and body weight. Height was measured to the nearest 0.01 cm with subjects standing without shoes. Weight was measured using a digital scale with subjects wearing light clothing and recorded to the nearest 0.1 kg. Body mass index (BMI) was calculated as body weight in kilograms divided by standing height in meters squared [7,37]. Those who had smoked more than 100 cigarettes in lifetime were defined as smokers; otherwise, they were defined as nonsmokers. Subjects were considered hypertensive as blood pressure ≥140/90 mmHg or they were treated with antihypertensive medications. Diabetes mellitus was defined either by the World Health Organization criteria or by self-report of being previously diagnosed as diabetes [38]. Family history was positive if first-degree relatives had CHD [39]. Fasting glucose, total cholesterol, HDL cholesterol, LDL cholesterol, and triglyceride levels were assayed according to standard laboratory procedures in the Department of Clinical Laboratory at Union Hospital and Tongji Medical College.

### Definition of coronary heart disease

The diagnostic criteria for CHD cases included one of the followings: (1) presence of a stenosis > 50% in at least one of the major segments of coronary arteries (right coronary artery, left circumflex, or left anterior descending arteries) based on coronary angiography, which can be seen in more details in previous studies [6,40,41]; (2) according to the World Health Organization criteria in terms of elevated cardiac enzymes, changes in electrocardiography and clinical symptoms; (3) a documented history of coronary artery bypass graft or percutaneous coronary intervention. Patients with congenital heart disease, cardiomyopathy, or severe vascular disease were excluded. All control subjects were determined to be free of CHD and peripheral atherosclerotic arterial diseases according to medical history, clinical examinations and electrocardiography. The controls were recruited in a population-based survey and resided in the same communities as the cases.

### Genotyping

Venous blood samples were collected after a 12 h overnight fasting and were drawn within 2 vacuum (ethylenediamine tetraacetic acid, EDTA) anticoagulation tubes for plasma and DNA. The blood specimens were frozen in −80°C until assayed. Genomic DNA was isolated with a Puregene kit (Gentra Systems, Inc., Minneapolis, MN, USA).

Two SNPs rs11722228 (*SLC2A9*) and rs4148152 (*ABCG2*), which were associated with uric acid levels in our recent GWAS [32], were selected and genotyped with the Sequenom MassARRAY iPLEX platform (Sequenom, Inc. San Diego, CA, USA) in 384-well format. The call rate was 97.6% and 97.1% for rs11722228 and rs4148152, respectively. In addition, we re-genotyped 5% of the total samples and the concordance is 100%. Both SNPs were consistent with HWE (*P >* 0.05) except for a slight deviation of rs4148152 in controls (*P* = 0.02) (data not shown).

### Data analysis

Categorical variables were presented in percentages and compared by Chi-square analysis. Continuous variables were expressed in mean ± SD and compared by student’s *t*-test or analysis of variation (ANOVA) unless otherwise specified. We conducted logistic regression analysis to calculate adjusted ORs and their 95% CIs for CHD risk by different genotypes of these SNPs in the multivariate models. Multivariate model 1 included age, sex, smoking, drinking, and family history of CHD. Multivariate model 2 included the same set of variables in model 1 plus BMI. Based on the model 2, model 3 further included total cholesterol, triglyceride, and the history of hypertension and diabetes. Homozygous genotypes CC and AA were used as reference genotypes for rs11722228 and rs4148152, respectively. The interactions between the independent SNPs and the covariates such as age, sex, BMI, smoking, and drinking were tested by introducing the SNP × environmental factor terms into the multivariate logistical regression model. Simultaneously, general linear model was performed and *P* for trend was calculated to observe the distribution of several traditional CHD risk factors among different genotypes of both SNPs. A two-side *P* value of < 0.05 was considered statistically significant. All statistical analyses were performed by the statistical analysis software package SPSS 12.0.

## Results

### Baseline characteristics analysis between cases and controls

In the present case–control study, CHD controls (n = 1,146) were frequency matched for age and sex to cases (n = 1,146). Baseline characteristics of study individuals are shown in Table 1. Compared with the control group, CHD cases were more likely to have significantly higher BMI, systolic blood pressure, fasting glucose, and HDL cholesterol levels (all *P* < 0.05). However, the levels of TC and LDL cholesterol were significantly lower in cases than those in controls, which might be due to the intake of cholesterol-lowering medications in CHD cases. The percentage of past history of diabetes and hypertension, and family history of CHD in cases were dramatically higher in contrast to that in control subjects (all *P* < 0.01).

### Associations of uric acid levels related SNPs with CHD risk

As shown in Table 2, the CC, CT, and TT genotype frequency of SNP rs11722228 in controls was 52.7%, 40.3%, and 7.0%. It was 50.2%, 42.0%, and 7.8% in the CHD group, respectively. The frequency of the C and T allele was not significantly different between the CHD group and the control group. For the SNP rs4148152, the genotype frequency of AA, AG, and GG in the controls was 42.0%, 48.1%, and 9.9%. In cases it was 47.4%, 42.6%, and 10.0%, respectively. No significant difference of the genotype frequency of rs4148152 was observed between the CHD group and the control group. SNP rs11722228 was not significantly associated with CHD risk. In contrast, the rs4148152-AG genotype had a significantly decreased risk of CHD (age and sex adjusted OR = 0.78, 95% CI: 0.66-0.93; *P* = 0.006) and the association stayed significant in a multivariate model (adjusted OR = 0.80, 95% CI: 0.66-0.97; *P* = 0.019) adjustment for age, sex, smoking, drinking and family history of CHD (model 1). However, the association altered to null when BMI was introduced into the model (model 2). Subjects carried the G allele of rs4148152 had a decreased risk of CHD (AG + GG) (age and sex adjusted OR = 0.80, 95% CI: 0.68-0.95; *P* = 0.010). The results remained significant in model 1 (adjusted OR = 0.80, 95% CI: 0.67-0.96; *P* = 0.015) but changed to borderline significant in model 2 when BMI was introduced into the model (*P* = 0.062). Further adjustment for the remaining traditional risk factors including total cholesterol, triglyceride, and past history of hypertension and diabetes got the similar null results (*P* = 0.306).

**Table 2:** **Adjusted Odds Ratios and 95% Confidence Intervals for CHD risk by different genotypes of the uric acid related SNPs in CHD cases and controls**

**SNPs gene location**	**Genotypes**	**Cases n (%)**	**Controls n (%)**	**Age- and sex- adjusted**	***P*** **value**	**Multivariate model 1** ^**a**^	***P*** **value**	**Multivariate model 2** ^**b**^	***P*** **value**	**Multivariate model 3** ^**c**^	***P*** **value**
rs11722228	CC	564(50.2)	586(52.7)	1.00	-	1.00	-	1.00	-	1.00	-
*SLC2A9*	CT	472(42.0)	449(40.3)	1.09(0.92-1.30)	0.319	1.14(0.95-1.37)	0.175	1.08(0.89-1.31)	0.445	1.06(0.85-1.32)	0.611
4p16.1	TT	87(7.8)	78(7.0)	1.16(0.83-1.60)	0.388	1.19(0.84-1.69)	0.322	1.05(0.73-1.51)	0.801	1.27(0.84-1.93)	0.262
	CT+TT	559(49.8)	527(47.3)	1.10(0.93-1.30)	0.268	1.15(0.96-1.37)	0.135	1.07(0.89-1.29)	0.452	1.09(0.88-1.35)	0.433
rs4148152	AA	533(47.4)	463(42.0)	1.00	-	1.00	-	1.00	-	1.00	-
*ABCG2*	AG	479(42.6)	530(48.1)	0.78(0.66-0.93)	0.006	0.80(0.66-0.97)	0.019	0.85(0.70-1.03)	0.088	0.92(0.74-1.16)	0.483
4q22	GG	112(10.0)	109(9.9)	0.89(0.67-1.20)	0.445	0.81(0.59-1.11)	0.192	0.81(0.58-1.12)	0.200	0.77(0.53-1.13)	0.182
	AG+GG	591(52.6)	639(58.0)	0.80(0.68-0.95)	0.010	0.80(0.67-0.96)	0.015	0.84(0.70-1.01)	0.062	0.89(0.72-1.11)	0.306

^a^, adjusted for age (continuous), sex (male, female), smoking (yes/no), drinking (yes/no) and family history of CHD (yes/no).

^b^, adjusted for the same set of variables in model 1 plus BMI (continuous).

^c^, adjusted for the same set of variables in model 2 plus total cholesterol (continuous), triglyceride (continuous), and the history of hypertension and diabetes (yes/no).

### Interactions between uric acid levels related SNPs and the traditional factors on CHD risk

We further investigated the associations between these two SNPs and CHD risk stratified by several traditional CHD risk factors such as sex (male/female), BMI (<24 and ≥24 kg/m^2^), smoking status (yes/no), alcohol consumption status (yes/no). Table 3 shows the ORs with 95% CIs adjusting for other risk factors except for the stratified factor. Among subjects who did not consume alcohol, the G allele of rs4148152 showed a moderate protective effect (adjusted OR = 0.79, 95% CI: 0.63-0.99; *P* = 0.038), however, no significant interactions were observed between alcohol consumption and SNP of rs4148152 on CHD risk (*P* for interaction = 0.47). Similarly, no interactions were observed for the two SNPs and other covariates on CHD risk.

**Table 3:** **Stratified associations of SNPs rs11722228 and rs4148152 with CHD risk by CHD traditional risk factors**

	**rs11722228 ORs (95% CIs)**				**P**	**rs4148152 ORs (95% CIs)**				
**Variables**	**CC**	**CT**	**TT**	**CT+TT**	***P***	**AA**	**AG**	**GG**	**AG+GG**	***P***
Sex										
Male	1.00	1.07(0.86-1.33)	1.14(0.75-1.71)	1.08(0.87-1.33)	0.62	1.00	0.83(0.67-1.03)	0.83(0.58-1.21)	0.83(0.67-1.02)	0.86
Female	1.00	1.19(0.78-1.81)	0.77(0.34-1.75)	1.12(0.75-1.68)		1.00	0.82(0.54-1.26)	0.71(0.34-1.46)	0.80(0.53-1.20)	
BMI (kg/m^2^)										
<24	1.00	0.97(0.74-1.27)	1.14(0.68-1.91)	1.00(0.77-1.29)	0.55	1.00	0.82(0.62-1.07)	0.84(0.54-1.32)	0.82(0.64-1.07)	0.99
≥24	1.00	1.22(0.92-1.60)	1.01(0.61-1.68)	1.18(0.91-1.54)		1.00	0.86(0.65-1.14)	0.76(0.48-1.22)	0.84(0.65-1.10)	
Smoking										
Yes	1.00	1.16(0.91-1.49)	1.23(0.77-1.97)	1.17(0.92-1.49)	0.23	1.00	0.82(0.64-1.06)	0.78(0.51-1.19)	0.82(0.64-1.04)	0.77
No	1.00	1.01(0.74-1.37)	0.85(0.48-1.53)	0.98(0.73-1.32)		1.00	0.87(0.64-1.19)	0.86(0.51-1.45)	0.87(0.65-1.17)	
Drinking										
Yes	1.00	0.88(0.62-1.26)	0.94(0.47-1.88)	0.89(0.64-1.25)	0.35	1.00	1.00(0.71-1.43)	0.82(0.42-1.56)	0.97(0.69-1.37)	0.47
No	1.00	1.19(0.94-1.50)	1.09(0.71-1.67)	1.17(0.94-1.47)		1.00	0.79(0.63-1.00)	0.78(0.53-1.14)	0.79(0.63-0.99)^a^	

ORs and 95% CIs were obtained from logistic regression analyses with adjustment for age, sex, BMI, smoking, drinking and family history of CHD except for the stratified factor.

^a^, OR is significant at the 0.05 level.

*P*, for interactions of CHD traditional risk factors with rs11722228 and rs4148152.

### The distribution of the traditional factors among different genotypes of rs11722228 and rs4148152 in controls

We also examined the distribution of CHD traditional risk factors including BMI, blood pressure, fasting blood glucose, TC, TG, HDL cholesterol and LDL cholesterol among different genotypes of SNPs rs11722228 and rs4148152 in controls. As Additional file 1: Table S1 demonstrates, none of these factors showed statistically significant differences among different genotypes of either SNP.

## Discussion

It still remains to be determined whether serum uric acid is an independent risk factor of CHD risk. As Mendelian randomization indicates, genetic variants could serve as an instrument to explore the causal associations between the biomarkers and the risk of diseases [13,42]. In the present study, we conducted a case–control study and selected two uric acid related SNPs rs11722228 (*SLC2A9*) [18,22,26,28,29] and rs4148152 (*ABCG2*) [12,30,31], which were found in our previous GWAS [32], to explore the potential contributing association of serum uric acid levels and CHD risk in a Chinese population. However, no association was found between the two uric acid related SNPs with CHD risk, indicating that there might not be causal association between them. Further studies are warranted to validate these results.

Previous GWASs have identified *SLC2A9* and *ABCG2* loci to be positively associated with serum uric acid levels and gout [13,22,43]. *SLC2A9*, also known as *GLUT9* (glucose transporter type 9), is a glucose transporter and plays a significant role in maintaining glucose homeostasis. *SLC2A9* is a causative gene for renal hyperuricemia and plays a significant role in urate reabsorption on renal proximal tubular cells [22,26]. *ABCG2* is one of adenosine triphosphate (ATP) binding cassette family and expressed in kidney proximal tubule cellular membrane [8,44]. It transports purine nucleoside analogues, which resemble the molecular structure of uric acid and mediates urate excretion in the kidney.

Unfortunately, we did not find significant associations between the two variants and CHD risk. In addition, considering that the CHD traditional risk factors might modify these associations, we conducted stratification and interaction analysis but no significant interactions were found between these covariates and the two variants on CHD risk. The results indicated that the associations of the uric acid related variants with CHD risk were not modified by these CHD traditional risk factors and there might be no causal association between serum uric acid levels and CHD risk.

Several issues contributing to this null result should be noted. Firstly, only two independent uric acid related variants SNPs of rs11722228 and rs4148152 were selected to perform this association study and they explained only 1.03% and 1.09% of the total variation of serum uric acid levels, respectively [32]. Selection of more variants that explained more percentage of uric acid levels was warranted in further studies. Secondly, relatively small sample size in the present case–control study provided relatively weak power to examine this association. For example, our study had more than 80% power to examine variants with MAF = 0.3 and OR = 1.2 at two-side *P* < 0.05. However, the present study only had 32% power to detect variants with MAF = 0.3 and OR = 1.1 at *P* < 0.05 significant level. Further studies with larger sample size were needed to validate our results. Thirdly, the present study was conducted in Chinese Han population, further studies conducted in other populations were necessary.

## Conclusions

In summary, this study did not find significant association of uric acid related SNP rs11722228 in *SLC2A9* with the risk of CHD in a Chinese population. Subjects carried the G allele of rs4148152 in *ABCG2* locus had decreased CHD risk, however, this association altered to borderline significant when BMI and other traditional risk factors were introduced into the multivariable model. No significant interactions between the two SNPs and CHD related risk factors were observed. Studies with larger sample size in other populations and genotyping more variants related to uric acid levels were warranted in future studies.

## Additional file

Additional file 1: Table S1. The distribution of the covariates among different genotypes of rs11722228 and rs4148152 in controls.

## Abbreviations

CHD: Coronary heart disease
SNP: Single nucleotide polymorphism
GWAS: Genome-wide association studies
OR: Odds ratio
CI: Confidence interval
EDTA: Ethylenediamine tetraacetic acid
BMI: Body mass index
HDL: High-density lipoprotein
LDL: Low-density lipoprotein
TC: Total cholesterol
TG: Triglyceride
*SLC2A9*: Solute carrier family 2, facilitated glucose transporter member 9
*ABCG2*: ATP-binding cassette, sub-family G, member 2
