# Supplementary material for: Associations of the uric acid related genetic variants in SLC2A9 and ABCG2 loci with coronary heart disease risk
Source: BMC Genet. 2015 Jan 30;16(1):4. doi: 10.1186/s12863-015-0162-7 (PMC4314773; doi:10.1186/s12863-015-0162-7)
Supplement: Additional file 1: Table S1. — The distribution of the covariates among different genotypes of rs11722228 and rs4148152 in controls. [file 12863_2015_162_MOESM1_ESM.doc]

**Additional file 1: Table S1. The distribution of the covariates among different genotypes of rs11722228 and rs4148152 in controls**

| **Variables** | **rs11722228** | | | ***P* ^a^ trend** | **rs4148152** | | | | ***P* ^a^** **trend** |
| --- | --- | --- | --- | --- | --- | --- | --- | --- | --- |
|  | **CC** | **CT** | **TT** |  | **AA** | **AG** | | **GG** |  |
| BMI (kg/m^2^) | 23.70±3.12 | 23.78±3.49 | 24.11±3.42 | 0.34 | 23.81±3.44 | 23.67±3.10 | 23.84±3.55 | | 0.81 |
| SBP (mmHg) | 133.85±21.83 | 133.53±37.39 | 130.10±22.80 | 0.79 | 134.44±36.66 | 132.22±22.06 | 136.51±23.44 | | 0.84 |
| DBP (mmHg) | 82.30±10.91 | 81.84±11.76 | 80.95±10.72 | 0.38 | 82.09±11.02 | 81.78±11.33 | 83.08±12.09 | | 0.72 |
| FBG(mmol/L) | 5.18±1.58 | 5.27±1.77 | 5.00±1.68 | 0.92 | 5.23±1.87 | 5.17±1.44 | 5.23±1.61 | | 0.82 |
| TC (mmol/L) | 4.68±0.90 | 4.65±0.94 | 4.71±0.84 | 0.95 | 4.73±0.95 | 4.64±0.93 | 4.63±0.72 | | 0.12 |
| TG (mmol/L) | 1.58±1.17 | 1.65±1.49 | 1.51±1.05 | 0.88 | 1.62±1.36 | 1.62±1.31 | 1.52±1.06 | | 0.57 |
| HDL cholesterol (mmol/L) | 1.10±0.35 | 1.10±0.36 | 1.11±0.31 | 0.70 | 1.10±0.37 | 1.09±0.35 | 1.15±0.35 | | 0.66 |
| LDL cholesterol (mmol/L) | 2.72±0.80 | 2.68±0.80 | 2.80±0.82 | 0.87 | 2.72±0.85 | 2.67±0.77 | 2.80±0.72 | | 0.95 |

*P* ^aa^, trend between different genotypes of SNPs rs11722228 and rs4148152 was calculated by general linear model, with adjustment for sex and age.

SB SBP: systolic blood pressure; DBP: diastolic blood pressure; GLU: fasting blood glucose; TC: total cholesterol; TG: triglyceride.
